# Treatment outcomes of acute leukemia during pregnancy at the National Institute of Hematology and Blood Transfusion

**DOI:** 10.1016/j.lrr.2026.100595

**Published:** 2026-05-25

**Authors:** Bach Quoc Khanh, Nguyen Hong Son, Nguyen Quoc Nhat, Tran Thu Thuy, Nguyen Thi Mai Huong, Nguyen Thu Chang, Nguyen Ha Thanh

**Affiliations:** a National Institute of Hematology and Blood Transfusion, Hanoi, Vietnam; b Hanoi Medical University, Hanoi, Vietnam

**Keywords:** Pregnancy, Gestation, Acute leukemia (AL), Treatment outcomes, Chemotherapy, Vietnam

## Abstract

Managing acute leukemia during pregnancy poses clinical challenges requiring a balance between maternal treatment and fetal preservation. From 2019 to 2024, 22 pregnant women were diagnosed with acute leukemia, including 7 acute lymphoblastic leukemia, 15 cases of acute myeloid leukemia, of which 1 was acute promyelocytic leukemia. Diagnosis occurred in the first, second, and third trimesters in 5, 10, and 7 patients, respectively. Nine patients elected pregnancy termination, one had a spontaneous abortion, one had intrauterine fetal demise, and three experienced concurrent maternal and fetal death. Eight patients delivered via elective cesarean section, including one twin pregnancy, resulting in 9 live births. Four neonates (including one twin pregnancy) were exposed to in utero chemotherapy, with no observed congenital anomalies. Chemotherapy was administered to 11 patients, including 3 during pregnancy, all achieving temporary disease control. These findings provide real-world data to inform the management of acute leukemia during pregnancy.

## 1 Introduction

Acute leukemia (AL) is an aggressive hematologic malignancy marked by uncontrolled proliferation of immature hematopoietic cells. Its occurrence during pregnancy is rare, with an incidence of 1 in 75,000 to 100,000 pregnancies. Acute myeloid leukemia accounts for two-thirds of cases, and acute lymphoblastic leukemia for one-third [1].

The clinical presentation of AL during pregnancy may be subtle or overlap with normal gestational symptoms—such as fatigue, dizziness, pallor, or easy bruising—leading to potential delays in diagnosis. Prompt recognition, however, is critical, as untreated disease progression can have life-threatening consequences for both mother and fetus. Treating AL during pregnancy poses a complex dilemma: while maternal survival depends on prompt initiation of cytotoxic chemotherapy, fetal exposure—especially during the first trimester—carries a high risk of teratogenicity and fetal demise. In some situations, pregnancy termination may be necessary to enable urgent treatment.

Beyond clinical complexities, patients often face emotional and psychological distress. Optimal management demands a multidisciplinary team to balance maternal and fetal outcomes. However, literature remains limited, mostly comprising case reports and small series. To address this gap, we conducted a retrospective study titled “**Treatment Outcomes of Acute Leukemia During Pregnancy at the National Institute of Hematology and Blood Transfusion**,” aiming to evaluate treatment strategies, clinical challenges, and maternal–fetal outcomes.

## 2 Materials and methods

### 2.1 Study population and period

This retrospective case series included 22 pregnant women newly diagnosed with acute leukemia, based on the 2016 World Health Organization (WHO) classification. All patients were treated at the National Institute of Hematology and Blood Transfusion (NIHBT), Hanoi, Vietnam, between January 2019 and January 2024.

### 2.2 Inclusion criteria

Following approval by the Institutional Review Board, medical records were reviewed retrospectively through the NIHBT electronic system. Eligible patients met all of the following criteria:

• Diagnosed with acute leukemia during pregnancy, according to the 2016 WHO diagnostic criteria, which require at least 20% blast cells in the bone marrow or peripheral blood.
• Aged 18 years or older.
• Diagnosis made during pregnancy; patients diagnosed prior to conception or postpartum were excluded.

All procedures were performed in accordance with the ethical standards of the 1975 Declaration of Helsinki and its 2013 revision.

### 2.3 Data collection

The following data were extracted from patient records: maternal age at diagnosis, gestational age and trimester at diagnosis, peripheral blood counts at diagnosis, percentage of blast cells in bone marrow, cytogenetic and molecular findings, pregnancy outcomes (delivery or abortion), mode of delivery, neonatal condition, chemotherapy regimen administered, response to chemotherapy, and both maternal and fetal outcomes. The first trimester was defined as up to the end of the 12th gestational week, and the third trimester as after the 27th gestational week.

In this study, palliative care was defined as supportive management without disease-directed chemotherapy, aimed at symptom control rather than curative intent. This included blood product transfusions (red blood cells and platelets), anti-infective therapy (prophylactic or therapeutic), and intensive care support when required.

### 2.4 Statistical analysis

Descriptive statistics were used to summarize patient demographics, clinical characteristics, and outcomes. Categorical variables were expressed as frequencies and percentages, while continuous variables were presented as mean (range). Data analysis was performed using SPSS software version 22.0 (IBM Corp., Armonk, NY, USA). No imputation was conducted for missing data.

### 2.5 Ethical considerations

This study was approved by the Ethics Committee of the National Institute of Hematology and Blood Transfusion (Approval No 2548/QĐ-HHTM). Given the retrospective nature of the study and the use of anonymized data, the requirement for informed consent for research participation was waived.

However, all patients received comprehensive counseling at the time of diagnosis regarding the potential risks and benefits of available treatment options, particularly chemotherapy during pregnancy. Treatment decisions were made based on the patients’ informed choices after thorough discussions with the hematology team and in consultation with obstetricians.

## 3 Results

### 3.1 Baseline characteristics and pregnancy outcomes of the study cohort

We identified 22 pregnant patients diagnosed with acute leukemia. The median maternal age was 32 years (range: 22–49), and the mean gestational age at diagnosis was 20.8 weeks (range: 5–37). As shown in Table 1, diagnosis occurred in 5 cases (22.7%) during the first trimester, 10 (45.5%) during the second, and 7 (31.8%) during the third. Seven patients had acute lymphoblastic leukemia (ALL), and 15 had acute myeloid leukemia (AML), including one case of acute promyelocytic leukemia (APL). Fourteen patients (63.6%) had a history of prior pregnancy (Table 2, Table 3).

**Table 1:** Baseline characteristics and pregnancy outcomes of 22 pregnant patients diagnosed with acute leukemia.

Characteristics	Trimester in which AL was diagnosed			Total
	First trimester	Second trimester	Third trimester	
**Number**	5	10	7	22
**Disease**				
AML	4	6	4	14
APL a	0	0	1	1
ALL	1	4	2	7
**Previous pregnancy**				
Yes	3	6	5	14
No	2	4	2	8
**Termination of pregnancy**				
Therapeutic abortion	4	5	0	9
Spontaneous abortion	1	0	0	1
Intrauterine fetal demise	0	1	0	1
Cesarean sections	0	1	7	8
Death b	0	3	0	3
**Maternal treatment approach**				
Palliative care	2	6	3	11
Chemotherapy	3	4	4	11
Chemotherapy during pregnancy	0	1	2	3
**Fetal outcomes**				
Fetal death c	5	9	0	14
Preterm (<37 weeks)	0	2	3	5
Term (≥37 weeks)	0	0	4	4
Live births (clinically stable at birth)	0	2	7	9
Exposed in utero to chemotherapy d	0	2	2	4

AL: Acute leukemia; AML: Acute myeloid leukemia; ALL: Acute lymphoblastic leukemia; APL: Acute promyelocytic leukemia.

Notes:

a APL is a subtype of AML. In the main text and analysis, APL is included within the AML group.

b “Death” refers to cases of concurrent maternal and fetal death during the diagnostic period. All cases occurred prior to initiation of induction chemotherapy and were due to severe complications (septic shock, pneumonia, or intracranial hemorrhage).

c Fetal death includes termination, spontaneous abortion, intrauterine fetal demise, and fetal loss associated with maternal death.

d One patient had a twin pregnancy, accounting for four fetuses exposed to chemotherapy despite only three mothers receiving treatment during pregnancy.

**Table 2:** Treatment approaches among the study cohort (n = 22).

Treatment approach	n	Percentage (%)
**Pregnancy termination with palliative care**	5	22.7%
**Pregnancy termination with chemotherapy**	8	36.4%
**Pregnancy continuation with palliative care**	6	27.3%
**Pregnancy continuation with chemotherapy**	3	13.6%

**Table 3:** Maternal and fetal outcomes following chemotherapy during pregnancy.

**Patient No.**	**Age**	**AL type**	Gravida/Parity	Gestational milestones	No. of Fetuses	Chemotherapy during pregnancy regimen	Timing of delivery (weeks)	Neonatal data	Neonatal outcome	Postpartum treatment	Follow-up duration (months)	Maternal treatment outcome
1	39	ALL/B	G0P0	Dx 20 w (T2); Chemo at 21w	2 (IVF)	DOX, vincristine, methylprednisolone	34w	1500 / 1800 g; NICU: Yes	Clinically stable at birth	Not available	3.2	Discharged; lost to follow-up
2	24	ALL/B	G0P0	Dx 28 w (T3); Chemo at 29w	1	DOX, vincristine, methylprednisolone, 6-MP	35w	2800 g; NICU: No	Clinically stable at birth	Hyper-CVAD	7.2	Refractory
3	32	APL	G0P0	Dx 37 w (T3); Chemo at 37w	1	ATRA	37w	3200 g; NICU: No	Clinically stable at birth	3 + 7+ATRA	24	CR

AL: Acute leukemia; ALL: Acute lymphoblastic leukemia; APL: Acute promyelocytic leukemia; CR: Complete remission; ATRA: All-trans retinoic acid; 6-MP: 6-mercaptopurine; DOX: Doxorubicin; NICU: Neonatal intensive care unit; T2, T3: Second and third trimester, IVF: in vitro fertilization, Dx: Diagnosis; Chemo: Chemotherapy; w: weeks.

Pregnancy termination was performed in 9 patients - 4 in the first trimester and 5 in the second. One patient experienced spontaneous abortion, and another had intrauterine fetal demise. Elective cesarean section was carried out in 8 patients, mostly diagnosed in the third trimester; only one was diagnosed in the second trimester.

Concurrent maternal and fetal death occurred in three patients during the diagnostic period, all in the second trimester (see Table 1), prior to initiation of induction chemotherapy. The primary causes of death were severe infection (pneumonia and septic shock) and intracranial hemorrhage in the setting of disseminated intravascular coagulation. Specifically, one patient with acute lymphoblastic leukemia died of severe COVID-19 pneumonia leading to acute respiratory failure. Another patient with acute myeloid leukemia presented with disseminated intravascular coagulation and died of intracranial hemorrhage shortly after admission. The remaining patient, also with acute myeloid leukemia, developed severe pneumonia with suspected sepsis and died within one week of hospitalization.

Among the eight women who achieved successful deliveries, all underwent elective cesarean section. A total of nine neonates were born, including one case of twin pregnancy. All neonates were born without congenital anomalies and were clinically stable at birth. Of the nine births, five were preterm and four were full-term. Notably, four neonates were exposed to chemotherapy in utero, including two from a twin pregnancy in one patient**.**

### 3.2 Treatment approach

Chemotherapy was administered to 11 patients (50%). The most common treatment approach was pregnancy termination followed by chemotherapy, observed in 36.4% of cases. Conversely, continuation of pregnancy with concurrent chemotherapy was the least frequent strategy, applied in only 3 patients (13.6%).

**Fig. 1:**
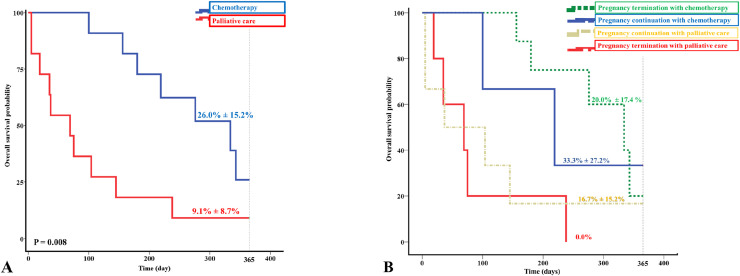
Kaplan–Meier survival analysis of 22 pregnant patients diagnosed with acute leukemia. (A) Comparison of 1-year overall survival between patients who received chemotherapy and those who received palliative care. Survival was significantly higher in the chemotherapy group (26.0% ± 15.2%) compared to the palliative care group (9.1% ± 8.7%) (p = 0.008). (B) One-year overall survival stratified by treatment strategy and pregnancy status. The highest survival rate was observed in patients who continued pregnancy and received chemotherapy (33.3% ± 27.2%), while no patients survived in the group that underwent pregnancy termination and received only palliative care (0.0%).

Fig. 1 presented Kaplan–Meier survival analyses for overall survival based on treatment approaches*.*

• Fig. 1A. Kaplan-Meier survival curve comparing overall survival (OS) between patients who received chemotherapy and those who received palliative care. The 1-year OS was 26.0% ± 15.2% in the chemotherapy group and 9.1% ± 8.7% in the palliative care group (p = 0.008).
• Fig. 1B Kaplan-Meier survival curve showing 1-year overall survival across four treatment strategies: termination of pregnancy with palliative care, termination with chemotherapy, pregnancy continuation with palliative care, and pregnancy continuation with chemotherapy. The 1-year overall survival (OS) was highest in the group that continued pregnancy and received chemotherapy (33.3% ± 27.2%) and lowest in the group that terminated pregnancy and received only palliative care (0.0%).

### 3.3 Treatment outcomes in patients who continued their pregnancy and received chemotherapy

Three patients continued their pregnancy while undergoing chemotherapy. Two patients, both diagnosed with ALL, received combination therapy consisting of doxorubicin (DOX), vincristine, methylprednisolone, and 6-mercaptopurine (6-MP). One patient with APL was treated with all-trans retinoic acid (ATRA) during pregnancy. All three patients underwent elective cesarean delivery between gestational weeks 34 and 37. Neonatal outcomes were favorable in all cases, with no congenital anomalies reported.

In terms of maternal outcomes, one patient achieved complete remission, one experienced refractory disease following delayed postpartum treatment, and one was lost to follow-up after discharge.

## 4 Discussion

Over a five-year period (2019–2024), we retrospectively analyzed 22 pregnant patients newly diagnosed with acute leukemia. Among them, 7 were diagnosed with ALL, and 15 with AML, including one case of APL, which was analyzed as part of the AML group. These results align with previous literature reporting that AML accounts for approximately two-thirds of acute leukemia cases during pregnancy [2,3]. In our cohort, 45.5% of patients were in the second trimester, while 22.7% and 31.8% were diagnosed during the first and third trimesters, respectively. Our findings are consistent with the study by Zhu et al. (2021) [4], who also identified the second trimester as the most common time for diagnosis, although other reports, such as that by Fardadfar N (2016) [5], found the highest rate in the first trimester. The mean gestational age at diagnosis in our cohort was 20.8 weeks, ranging from 5 to 37 weeks.

Regarding pregnancy outcomes, nine patients elected for pregnancy termination (4 in the first trimester and 5 in the second), while 1 experienced a spontaneous abortion and 1 had an intrauterine fetal demise. Additionally, 3 patients died during the diagnostic phase, resulting in concurrent maternal and fetal death—accounting for 13.6% of the cohort. Eight patients underwent elective cesarean delivery, primarily those diagnosed in the third trimester. A total of nine neonates were delivered, including one set of dizygotic twins. All neonates were born without congenital anomalies and were clinically stable at birth. Among them, five were born preterm and four at term. Notably, all four neonates with documented in utero exposure to maternal chemotherapeutic agents were born without any congenital malformations.

Our findings are consistent with prior studies that reported similar pregnancy outcomes in patients with acute leukemia. Zhu et al. (2021), in a study of 21 pregnant patients diagnosed with acute leukemia, reported 6 terminations, 1 spontaneous abortion, 9 live births, and 5 early maternal deaths. Four neonates were exposed to chemotherapy in utero, yet none exhibited congenital anomalies [4]. Similarly, in a French multicenter study by Youcel Chelghoum et al. (2005), 23 of 37 pregnant patients with acute leukemia delivered infants without congenital anomalies, including 15 who had been prenatally exposed to chemotherapeutic agents [6].

Regarding long-term outcomes, available studies—though limited in number—offer reassuring evidence. Avilés and Neri followed 84 children with prenatal exposure to chemotherapy over a mean period of 18.7 years and found no cognitive, neurological, or psychological abnormalities. Additionally, normal fertility and cognitive development were reported in 12 children of the second generation [7]. Likewise, Elyce H. Cardonick's research demonstrated no significant differences in cognitive skills, academic achievement, or behavioral performance between children exposed to chemotherapy in utero and those who were not [8].

Taken together, these results, both from our cohort and international studies, support the growing body of evidence suggesting that in utero exposure to chemotherapeutic agents—when administered during the second and third trimesters—does not necessarily result in adverse short- or long-term outcomes for the neonate.

### 4.1 Overall outcomes

In our cohort, 50% of patients did not receive chemotherapy, reflecting ongoing challenges in initiating treatment during pregnancy. One-year OS was significantly higher in patients receiving chemotherapy (26.0% ± 15.2%) versus palliative care (9.1% ± 8.7%, *p* = 0.008). Further subgroup analysis (Fig. 1B) demonstrated that survival varied according to treatment strategy and pregnancy status. By treatment and pregnancy status, the one-year OS was 33.3% ± 27.2% in patients who continued pregnancy and received chemotherapy, and 20.0% ± 17.4% in those who terminated pregnancy and received chemotherapy. For patients who continued pregnancy with palliative care, one-year OS was 16.7% ± 15.2%. Notably, no patients who terminated pregnancy and received only palliative care survived beyond one year (OS = 0.0%). Although not statistically significant due to small sample size, these trends suggest chemotherapy may improve maternal outcomes, even during pregnancy. This is consistent with recent studies on acute leukemia in pregnancy. Maggen et al. (2022) emphasized that chemotherapy can be safely administered after the first trimester without requiring pregnancy termination [9]. A retrospective study by Wang et al. (2021) involving 52 pregnant women with acute leukemia found that timely chemotherapy during pregnancy did not significantly compromise maternal or fetal outcomes, with all newborns alive and without deformities [10]. These findings support that, with appropriate timing and multidisciplinary care, chemotherapy during pregnancy appears to be feasible and potentially beneficial in selected cases.

### 4.2 Management of leukemia in first trimester

Five patients were diagnosed with acute leukemia during the first trimester, including four with AML and one with ALL. All five elected to terminate the pregnancy early. Among them, three patients (all with AML) subsequently received chemotherapy.

During the first trimester, the likelihood of achieving a successful pregnancy outcome is extremely low due to the high teratogenic risk associated with chemotherapy. In our study, early pregnancy termination was prioritized in accordance with current international guidelines, enabling prompt initiation of treatment. Without timely intervention, maternal death may occur within weeks to months of diagnosis [2].

### 4.3 Management of leukemia in second trimester

Ten patients were diagnosed with acute leukemia during the second trimester (6 AML, 4 ALL). Six (60%) underwent pregnancy termination, and three died early along with fetal loss. Chemotherapy was given to four patients; only one continued pregnancy during treatment and later delivered via cesarean at 34 weeks (**Case No. 1)**. This case involved a 39-year-old Laotian woman with dichorionic twins conceived via in vitro fertilization (IVF), treated with DOX, vincristine, and methylprednisolone. Both twins were delivered healthy, with no anomalies, and the patient subsequently returned to her home country, resulting in limited follow-up data. Although the twins in Case 1 were admitted to the neonatal intensive care unit (NICU) due to prematurity (birth weights: 1500 g and 1800 g), they were clinically stable and had no congenital anomalies. Therefore, the neonatal outcome was considered healthy. These findings suggest that pregnancy termination is often preferred in second-trimester diagnoses. However, in selected cases, continuing pregnancy with chemotherapy can be safe and beneficial. This case highlights the importance of individualized care and the potential for maternal and fetal benefit using second-trimester-compatible chemotherapy.

### 4.4 Management of leukemia in third trimester

Seven patients were diagnosed with acute leukemia during the third trimester of pregnancy. This period is considered relatively safe for the fetus, as the teratogenic risk of chemotherapeutic agents tends to decline as gestation nears term[6]. However, chemotherapy during late pregnancy still requires careful monitoring due to the potential for treatment-induced cytopenias and spontaneous labor. Most guidelines recommend avoiding chemotherapy after 35 weeks of gestation to minimize risks of maternal hematologic complications during labor [11]. In our study, chemotherapy was delayed in these cases, with careful timing of delivery determined through multidisciplinary consultation. In our cohort, 4 out of 7 patients diagnosed in the third trimester received chemotherapy, including two who continued their pregnancies while undergoing treatment (one with ALL and one with APL).

**The patient in Case No. 2** was diagnosed with ALL at 28 weeks of gestation. Due to marked hyperleukocytosis (white blood cell count 249 × 10⁹/L), prompt initiation of chemotherapy during pregnancy was necessary to prevent leukostasis and disease progression. A regimen consisting of DOX, vincristine, methylprednisolone, and 6-mercaptopurine was administered during pregnancy. In parallel, a multidisciplinary consultation with obstetricians was conducted to determine the optimal timing for delivery. After delivery at 35 weeks of gestation with a clinically stable neonate, the patient elected to delay postpartum chemotherapy for more than three months in order to spend additional time with her newborn, despite being fully counseled regarding the potential risks of treatment delay. During this period, no definitive anti-leukemic therapy was administered, and the disease subsequently progressed, resulting in refractory status after re-initiation of treatment.

**The patient in Case No. 3** was diagnosed with acute promyelocytic leukemia (APL) and concurrent disseminated intravascular coagulation (DIC score: 5) at 37 weeks of gestation. This was her first pregnancy and was classified as high risk with poor maternal and fetal prognosis. APL is considered a hematologic emergency. Managing APL during pregnancy poses a significant challenge due to frequent coagulopathy, which can complicate pregnancy and delivery. Furthermore, the teratogenic risks associated with ATRA and ATO necessitate careful consideration. Recent guidelines provide specific recommendations for managing APL in pregnancy [12]. After obtaining informed consent, she was started on ATRA monotherapy and corticosteroids, with close monitoring of clinical and coagulation parameters. Once coagulopathy was temporarily stabilized, she underwent emergency cesarean section at our center. Postpartum, she received a standard “3 + 7+ATRA” chemotherapy regimen. Both the mother and the newborn remained clinically stable and in good condition. This was the only APL case in our cohort and was successfully managed.

## 5 Conclusion

Between 2019 and 2024, we managed 22 cases of acute leukemia during pregnancy (7 ALL, 15 AML, including 1 APL). Nine patients opted for pregnancy termination, while one had a spontaneous abortion and one experienced intrauterine fetal demise. Eight patients underwent planned cesarean delivery, resulting in nine healthy live births without congenital anomalies.

Eleven patients received chemotherapy, including three treated during pregnancy—two in the third trimester and one in the second. All three achieved temporary disease control and maintained pregnancy until viability, suggesting that chemotherapy during pregnancy may be feasible in selected cases under close multidisciplinary monitoring.

Importantly, all successful cases of chemotherapy during pregnancy involved close collaboration between hematology and obstetrics, which was crucial for balancing maternal treatment with fetal considerations. While our findings suggest that chemotherapy can be administered during pregnancy in selected cases, the small sample size and heterogeneity of treatment approaches warrant cautious interpretation and highlight the need for larger studies.

## Informed consent

This study was approved by the Ethics Committee of the National Institute of Hematology and Blood Transfusion (Approval No. 2548/QĐ-HHTM). Given the retrospective nature of the study and the use of anonymized data, the requirement for informed consent for research participation was waived.

However, all patients received comprehensive counseling at the time of diagnosis regarding the potential risks and benefits of available treatment options, particularly chemotherapy during pregnancy. Treatment decisions were made based on the patients’ informed choices after thorough discussions with the hematology team and in consultation with obstetricians.

## Funding

No external funding was received for this study

## Conflict of interest

The authors declare no conflict of interest.

## CRediT authorship contribution statement

**Bach Quoc Khanh:** Writing – review & editing, Supervision, Project administration, Methodology, Conceptualization. **Nguyen Hong Son:** Writing – original draft, Writing – review & editing, Methodology, Formal analysis, Data curation, Conceptualization. **Nguyen Quoc Nhat:** Supervision, Writing – review & editing. **Tran Thu Thuy:** Supervision, Writing – review & editing. **Nguyen Thi Mai Huong:** Data curation, Investigation. **Nguyen Thu Chang:** Resources, Investigation. **Nguyen Ha Thanh:** Supervision, Writing – review & editing.

## Declaration of competing interest

The authors declare that they have no conflicts of interest related to this study.
